# Medical Opinion and Movement

**Published:** 1906-09-15

**Authors:** 


### Medical Opinion and Movement.

A medical practitioner of great experience and influence has formed the conclusion that the use of American flour, which is said to be often adulterated with pounce, may be a frequent cause of appendicitis. This flour is largely used in making white bread, and the cases which have come under notice have been confined to eaters of white bread. This may be a good reason for substituting brown for white bread in the household, but there are other reasons too. Whole wheat-meal contains much greater quantities of phosphates than the best white flour, and the demand for brown bread amongst poor families should be encouraged by every medical officer and all who have it in their power to make the facts known and understood by the people generally.

We are glad to note that educationists and those interested in temperance are taking steps to organise a series of lectures on hygiene and temperance, through the London School of Economics and the Birkbeck Institute, during the autumn and winter, for the instruction of teachers of children especially. The chairman of the Advisory Board of Hygiene and Temperance explains that the syllabus used will be practically that drawn up by a Committee of the medical profession. At present few teachers are qualified to teach children these subjects in an unprejudicial, practical, and sober manner. We wish all success to this movement, for in this matter American teachers and schools are far in advance of those in this country.

The War Office as reorganised does not show signs of the improvement hoped for in consequence of new men and new measures. The outbreak of typhoid fever in the officers' mess at Fleetwood Camp, whereby three officers lost their lives, is not only regrettable in itself, but casts a serious responsibility on those in charge of the arrangements. Three causes have been suggested, and the searching inquiry about to take place will be awaited with painful interest. These causes are water supply; but as that supplied to the camp was the same as the town supply, and Fleetwood is free from typhoid, a dirty water-cart is put down as the possible origin of this source. Surgeon-General Evatt points out that the condition of officers' messes is often wretchedly bad. He has frequently found them the dirtiest places in the barracks in this country, and in India they are even worse. "North Lancashire Colonel" attributes the probable cause to the fact that usually all the arrangements and supplies for the officers' mess are controlled by private contractors who are generally free from efficient control. The handling of this case should prove a practical test of the power for good which the strong man as War Secretary, Mr. Haldane, can or cannot exercise.

The sixtieth Report of the Commissioners in Lunacy shows that this branch of inspectorial work proceeds with a dull monotony of non-resultant routine. It may well be asked by the taxpayers, "Can these dry bones live?" Not until the Press and the public cease to treat asylums and lunatics as dust-heap products, without interest, devoid of use, and incapable of improvement. The dust-heap attitude of mind is common in England, and, despite the attacks upon waste and extravagances in other directions, the English nation permits the wasteful expenditure of millions of money without a protest where the feeling exists that it is an outlay on necessary scavenging. So the Poor-law, Prisons and Crime, and Lunacy expenditure grows and ramifies; and the public pay, whilst waste and unnecessary expenditure multiply continuously as the years roll by. New buildings are being put up every year when existing buildings would be saved from decay by being devoted, as they well might be, to the uses to which the new buildings will be put. In other words, the capitation grant ought to be withheld in the case of chronic dements who could be properly and economically provided for in the workhouses.

The actual results shown by the Report, so far as mere numbers are concerned, are encouraging so far as they go. Although the patients under treatment on January 1, 1906 (121,979), were 2,150 in excess of the previous year's figures, the average increase for ten years ended 1905 was 2,554, and for five years 2,807. So that the increase for the year 1905 was less by some hundreds than the average increase for the decennium and also for the quinquennium. Of the total number of insane patients under treatment, 73.2 per cent. were maintained in county and borough asylums, which is not surprising, seeing that 91.2 per cent. of the certified insane are paupers. Only about a three-hundredth part of the paupers insane in the Metropolis are confined in workhouses. Why the Scotch system in this matter is not followed in the case of chronic dements in London and the ratepayers' pockets so protected is a mystery. Surely some of our County Councillors might strive to solve and to remedy this extravagance with advantage to the public and patients and for the credit of their own reputation. The Commissioners fight rather shy of the causes which conduce to insanity. "Morbid material changes due to pain or defects in brain structure"; "others which cannot (in the present state of knowledge) be assigned any material basis"; emotion where the brain is initially feeble, congenital defects, and the natural decay of cerebral functions with advancing years are mentioned in this connection. Premature decay of mind and body from excesses and over-strain are sometimes recognised as "old age" causes, whereas justice and science demand a more accurate classification. The Commissioners urge one point worthy of attention, which will never probably be enforced unless the taxpayer awakes and demands economy and financial method in the management of these institutions. The Commissioners urge an increase in their number; but, much as we are in favour of this reform we cannot support it unless guarantees are first forthcoming that additional Commissioners will mean modern and improved methods of administration, of inspection, of financial management and economical administration.

